# Emerging Roles of Circular RNAs in Thyroid Cancer

**DOI:** 10.3389/fcell.2021.636838

**Published:** 2021-04-26

**Authors:** Fada Xia, Zeyu Zhang, Xinying Li

**Affiliations:** Department of Thyroid Surgery, Xiangya Hospital, Central South University, Changsha, China

**Keywords:** thyroid cancer, circular RNA, cell signaling, diagnosis, prognosis

## Abstract

Thyroid cancer (TC) has the highest incidence among endocrine malignancies. Thus, it is essential to achieve a deep understanding of various mechanisms of development and progression of TC. circRNAs are recognized by multiple studies as being dysregulated in TC. Accumulating evidences have revealed that circRNAs serve as regulatory molecules involved in various biological processes in TC, including cell proliferation, apoptosis, invasion/migration, metabolism, and chemoresistance. Furthermore, circRNA can also serve as an effective tool in TC researches of diagnosis, prognosis, and treatments. Thus, this review is to outline the characteristics of circRNAs, generalize their categories and functions, and highlight the expression of circRNAs in TC. Meanwhile, we are expecting to achieve a comprehensive understanding of new therapies based on circRNAs in treating or preventing TC.

## Introduction

As the most common cancer of endocrine system, thyroid cancer (TC) incidence has been increasing faster than any other cancer type since the 1990s. However, it has been referred as over-treatment due to the low risk, non-lethal characteristics of TC that are often incidentally detected from a large subclinical reservoir of disease (Roman et al., 2017). According to estimation, TC would become the fourth leading cancer diagnosis during the next 10 years (Rusinek et al., 1817). TC can usually be classified into papillary thyroid carcinoma (PTC), follicular thyroid carcinoma (FTC), anaplastic thyroid carcinoma (ATC), and medullary thyroid carcinoma (MTC). Among these four types of TC, PTC and FTC are defined as differentiated thyroid cancers (DTCs), with incidence rates over 90%. The majority of DTCs have an indolent behavior, but with a significantly high proportion of patients suffering from lymph node metastasis (Asa and Ezzat, 2017). Surgery is the most common treatment of DTC, even in cases with local and distant metastases. MTC comprises 5% to 10% of all TC. Total thyroidectomy and lymph dissection can result in a biochemical cure (normalization of calcitonin and CEA) for only 40% of the time. Even when a biochemical cure is achieved, approximately 9% of patients will later develop recurrent (Kloos et al., 2009; Maxwell et al., 2014). ATC, which accounts for less than 2% of TC, contributes to the most mortality of TC. Patients with ATC usually have a relatively poor prognosis with the median survival less than a year, mainly due to the resistance to common TC therapies (Hsu et al., 2014). Given the past 30 years of researches on molecular mechanisms, TC is proved to be facilitated by various dysregulated genes via multiple pathways. Presently, 90–95% of TC can be explained by the MAPK, PI3K/Akt, and other known pathways; however, the associated mechanisms of the remaining 5–10% are poorly recognized (Murugan et al., 2018). Therefore, in-depth understanding of the molecular basis and signaling pathways related to TC proliferation and progression not only is expected to clarify the mechanism of TC development and provide molecular biomarkers for TC diagnostic and prognostic prediction, but also has a profound impact on molecular targeted therapy.

The untranslated transcripts, also called non-coding RNAs (ncRNAs), account for 97% of human genome, which can be classified into short (19–31 nucleotides), mid (20–200 nucleotides), and long (>200 nucleotides) based on their length. Currently, many microRNAs (miRNAs) and long-ncRNAs (lncRNAs) are investigated by a great number of studies (Grillone et al., 2020). Meanwhile, circular RNA (circRNA) is a large class of non-coding RNA containing a unique covalent loop structure without 5′-cap and 3′-poly (A) structures, causing their resistance to exonuclease degradation, which mainly depends on the 5′ and 3′termini (Li C. et al., 2020). Due to the advancing sequencing technologies and bioinformatics, novel circRNAs are continuously identified with their specific expression patterns in different tumor stages and characteristics. Along with their conservation, circRNA may be involved in various types of tumor being as a special biomarker (Meng et al., 2017).

At present, many circRNAs are found being differentially expressed with significant functions in multiple diseases (Xia et al., 2020). Recent evidences also showed the regulatory role of circRNAs in cervical (Chaichian et al., 2020), lung (Chen Y. et al., 2018), oral (Cristóbal et al., 2020), gastric (Fang et al., 2019), and breast cancer (Jahani et al., 2020). Moreover, there are studies reporting that circRNAs were dysregulated in TC. Thus, this review was focused on the biogenesis and function of circRNA, the dysregulated expression of circRNAs in TC, and their involvement in TC development. In addition, we also addressed the potential role of circRNAs as diagnostic and prognostic biomarkers, as well as therapeutic targets for TC.

## Biogenesis and Function of circRNA

### Synthesis and Biological Characteristics of circRNAs

**FIGURE 1:**
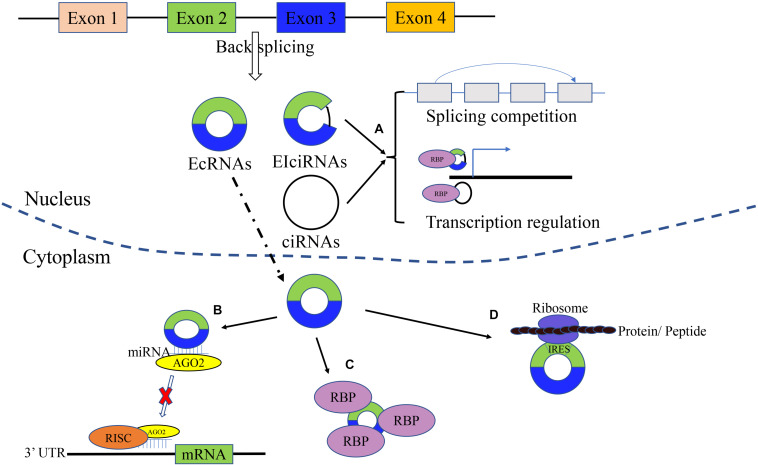
Biogenesis and molecular function of circRNAs. circRNAs are generated from exonic and/or intronic sequences of primary transcripts by back-splicing. The main types of circRNAs includes EcRNAs, EIciRNAs, and ciRNAs. **(A)** Gene translation regulation. circRNA can regulate related downstream gene and parental gene transcription. **(B)** microRNA sponge. circRNAs can act as microRNA sponges and suppress the effects of microRNA on target genes. **(C)** Binding RBP. By interaction with RBP, circRNAs can inhibit/enhance protein function, and promote the colocalization of protein. **(D)** Translation function. circRNA can be translated into protein/peptides.

As shown in Figure 1, circRNA is a novel class of noncoding RNAs with a covalently closed loop, generating from exonic and/or intronic sequences of primary transcripts by back-splicing (Cristóbal et al., 2020). There are four types of circRNAs: exonic circRNAs or ecircRNAs (the most frequent) that arise from exons, ciRNAs that originate from introns, EIciRNAs that arise from both exons and introns, and infrequent tricRNAs produced from transfer RNA introns (Chaichian et al., 2020). The circularization of ecircRNA or EIciRNA is mainly recognized as Lariat-driven, intron pairing-driven, and RNA-binding protein (RBP)-dependent, while ciRNA formation is based on conserved sequences near the spliceosome (Yang X. et al., 2020). Intronic circRNAs are dependent on groups I/II ribozymes, while the exonic circRNAs are dependent on a spliceosomal ribozyme (Jahani et al., 2020). There are several factors that have an impact on the biosynthetic speed (Fang et al., 2019). For instance, RBPs can activate and inhibit the formation of circRNA. Meanwhile, the biosynthesis of circRNA can be enhanced by muscleblind-like (MBL) protein and quaking (QKI). In addition, ADAR1 plays an inhibitory role in circRNA formation (Ma et al., 2019; Jahani et al., 2020). The characteristics of circRNAs are as follows: conservative, stable, highly expressed and tissue-specifically expressed (Hao et al., 2019). Compared with linear RNAs, circRNAs are not sensitive to RNases (Hao et al., 2019). Most of the circRNAs are located in the cytoplasm (ecircRNAs), while ciRNAs and EIciRNAs are located in the nucleus (Cristóbal et al., 2020).

### circRNAs act as miRNA Sponges

miRNAs play the post-transcriptional regulation with target genes in which it is referred to as miRNA response elements (MREs). A single miRNA is able to target multiple genes with consistent MREs, thus describing the so-called competing endogenous RNA (ceRNA) network (Limb et al., 2020; Lin et al., 2020). Currently, the research of ceRNAs’ effects covers a wide range of subjects, involving many ncRNAs, including lncRNA, circRNAs, pseudogenes, and viral transcripts (Lin et al., 2020). miRNA sponging is presently recognized as the main mechanism that circRNAs are involved in (Limb et al., 2020). Many circRNA–miRNA–mRNA axes have been reported to be associated with the development of malignant diseases. For instance, CiRS-7 possesses miR-7 binding sites, thus sponging miR-7. Studies also showed that CiRS-7 was associated with cell proliferation and progression by sponging miR-7 to regulate the relative pathways in various cancers (Zheng et al., 2017; Pan et al., 2018; Yang X. et al., 2020). Moreover, CiRS-7 is an effective biomarker in diagnosis and predicting prognosis, which can help clinical decisions toward solid tumors (Zou et al., 2020). Meantime, circRNA homeodomain-interacting protein kinase 3 (CircHIPK3) is associated with various human cancers. There are numerous researches indicating that circHIPK3 functions as a miRNA sponge (miR-124, miR-149, miR-29b, and miR-637, etc.) to regulate the target genes and exert specific biological effects, including regulation of cell proliferation, invasion, and migration (Chen et al., 2020; Zhang Y. et al., 2020).

### circRNAs Can Interact With Proteins

Bioinformatics analyses of circRNA sequences can be used to predict binding sites for RBPs, while RNA pull-down and RNA immunoprecipitation (RIP) are the most used techniques to verify the binding between circRNAs and proteins (Xie et al., 2020). circFOXK2 promotes pancreatic ductal adenocarcinoma cell growth, migration, invasion, and apoptosis via sponging miR-942, eventually promoting the expression of several downstream genes. Furthermore, a circRNA pulldown identifies 94 types of proteins interacting with circFOXK2, with their functions ranging from cell adhesion to structural molecule activity. Specifically, circFOKX2 increases the expression of oncogenes via interacting with YBX1 and hnRNPK (Wong et al., 2020). On the other hand, HuR, as a well-studied RBP, can mediate the expression of proteins via binding with a wide range of RNAs. The binding between CircPABPN1 and HuR can lower the expression of PABPN1, providing an example of competition between a circRNA and its cognate mRNA for an RBP that affects translation (Abdelmohsen et al., 2017).

### circRNAs Regulate Gene Transcription

The processes of producing circRNAs can influence the biosynthesis of mRNAs. Ci-ankrd52 is related to the extension mechanism of RNA pol II, while circRNA acts as a positive regulator of RNA pol II transcription, indicating the cis-regulation of non-coding introns on the transcription of their parent genes (Zhang et al., 2013; Khanipouyani et al., 2020; Zhang X. et al., 2020). In comparison to ecircRNAs, EIcircRNAs and ciRNAs are mainly enriched in the nucleus, which can mediate the biosynthesis of protein by transcriptional or post-transcriptional regulation (Khanipouyani et al., 2020; Zhang X. et al., 2020). In addition, EIciRNA enhances the expression of its parent gene, highlighting a regulatory strategy for transcriptional control by the specific interaction between U1 snRNA and EIciRNA (Li et al., 2015).

### circRNA Can Be Translated Into Protein

circRNAs are presently recognized as cannot be translated due to the loop structure and lack of polyadenylated tail and internal ribosome entry site (IRES) (Yang X. et al., 2020). Intriguingly, some circRNAs that contain the IRES can be translated, which may be triggered under certain conditions (Zhou et al., 2019; Zhang X. et al., 2020). It has been demonstrated that circRNA can be translated by inserting an IRES into a synthetic circRNA (Chen and Sarnow, 1995; Yang X. et al., 2020). Additionally, circ-ZNF609 contains an open reading frame and associates with heavy polysomes, which can be translated in a splicing-dependent and cap-independent manner (Legnini et al., 2017).

Generally, acting as a miRNA sponge is the most studied function with great potential in circRNA-related researches. While studies focusing on other functions of circRNA are also welcomed, we have a very positive view on applying circRNA-related therapies to clinical practice based on the function as a miRNA sponge. Furthermore, the relationships between these various functions are little known.

### Expression Profiles of circRNAs in TC

Several studies have investigated the circRNA expression profiles in TC by using microarray profiling and high-throughput RNA sequencing, showing that many dysregulated circRNAs can be related to TC. Peng et al. (2017) have firstly profiled the circRNA expression of PTC tumors. Ninety-eight dysregulated circRNAs were identified between PTC and adjacent normal tissue, while 355 were identified between PTC and benign thyroid lesions (Peng et al., 2017). Lan et al. (2018b) also investigated and validated the circRNA profiles of PTC by RNA-Seq and qRT-PCR. Eighty-seven differentially expressed circRNAs were identified with 33 novel circRNAs (Lan et al., 2018b). Moreover, Ye et al. (2020) identified 358 novel circRNAs using five matched PTC and adjacent normal tissues. Yao et al. (2019) selected 12 samples, consisting of four invasive tumor tissues, four non-invasive tumor tissues, and four matching normal tissues for circRNA microarray analysis revealing 607 differentially expressed circRNAs between invasive tumor tissues and adjacent normal tissues, while 49 between invasive and non-invasive tumor tissues. The intersection of these two sets contained 13 circRNAs (Yao et al., 2019). Teng et al. (2018) reported a comprehensive transcriptome-wide analysis by using RNA sequencing of 11 pair PTC and normal tissues (the largest series as reported). They identified a total of 17,864 circRNAs in thyroid tissues and 146 dysregulated circRNAs from 128 host genes (Teng et al., 2018). The dysregulated circRNAs identified by sequencing and microarray data are listed in Table 1. It should be noted that many clinical and analytical factors can affect the final results of sequencing data (Li R. et al., 2020).

**TABLE 1:** Overview of circRNAs identified by microarrays and RNA sequencing in TC.

Study	Sample	Special treatment	Detection method	Number of circRNAs	Number of differently expressed circRNA (>two fold change)	Number of novel circRNA*	References
1	6 paired TC and normal tissues	–	Microarray	5490	98 (88 upregulated, 10 downregulated)	–	Peng et al., 2017
	6 paired TC and benign nodule tissues	–	Microarray	5490	355 (129 upregulated, 226 downregulated)	–	Peng et al., 2017
2	3 paired TC and normal tissues	–	RNA sequencing	9103	87 (41 upregulated, 46 downregulated)	33	Lan et al., 2018b
3	3 paired TC and normal tissues	Rnase R**	Microarray	13,617	383 (206 upregulated, 177 downregulated)	–	Ren et al., 2018
4	5 paired TC and normal tissues	Rnase R	RNA sequencing	NA	231 (133 upregulated, 98 downregulated)	–	Sun J. W. et al., 2020
5	5 paired TC and normal tissues	Rnase R	Microarray	NA	1137 (678 upregulated, 459 downregulated)	358	Ye et al., 2020
6	5 paired invasive TC and normal tissues	–	Microarray	NA	607***	–	Yao et al., 2019
	5 paired invasive and non-invasive TC tissues	–	Microarray	NA	49***	–	Yao et al., 2019
7	5 paired TC and normal tissues	Rnase R	Microarray	NA	924 (478 upregulated, 446 downregulated)	–	Wu et al., 2020a
8	Serum exosomes from 3 paired PTC patients and control group	Removed ribosomal RNA, Rnase R	RNA sequencing	NA	22 (3 upregulated, 19 downregulated)	–	Yang et al., 2019
9	11 paired TC and normal tissues	–	RNA sequencing	17,864	146***	–	Teng et al., 2018
10	5 paired TC and normal tissues	Removed ribosomal RNA, Rnase R	RNA sequencing	NA	54 (35 upregulated, 19 downregulated)	–	Liu et al., 2020b
11	3 paired TC and normal tissues	Removed ribosomal RNA, Rnase R	RNA sequencing	30,954	28 (17 upregulated, 11 downregulated)	–	Long et al., 2020

*circRNAs that have not been annotated at the time the studies were ready for publication. **Linear RNAs were removed from total RNAs using RNase R. ***The number of upregulated and downregulated circRNA was not revealed in this study. NA, not available.

According to the circRNA expression profiles in PTC, a large number of aberrantly expressed circRNAs are shown in TC tissues; some of these circRNAs are upregulated and serve as oncogenes, while the downregulated one serves as a tumor suppressor. The number of upregulated and downregulated circRNAs identified by microarray profiling and high-throughput RNA sequencing shows no difference in tendency. Researches about the function of circRNAs involved in TC mainly focus on upregulated circRNA, while only a few studies focus on downregulated circRNAs, for instance, circITCH (Wang et al., 2018) and hsa_circ_0007694 (Long et al., 2020). RNA FISH and cell fraction assay are commonly used to locate circRNAs in the TC cells. FISH results demonstrated that circNUP214 (Li et al., 2018), circBACH2 (Cai et al., 2019), circRAPGEF5 (Liu et al., 2019), and circ_0067934 (Zhang et al., 2019) were predominantly localized in the cytoplasm. The cell fraction assay and FISH analysis results demonstrated that circFNDC3B (Wu et al., 2020b), circ-0005273 (Zhang W. et al., 2020), and circFOXM1 (Ye et al., 2020) were predominantly localized in the cytoplasm, while hsa_circ_0058124 was shown in both nucleus and cytoplasm (Yao et al., 2019). Meanwhile, circRNA_102171 was proven to be predominantly located in the nucleus (Bi et al., 2018). The difference of circRNA in cellular localization indicates that circRNA may be involved in a wide range of biological functions through different mechanisms. Despite this large amount of dysregulated circRNAs discovered in TC, there is still a long way to go investigating their biological mechanisms in TC.

### circRNAs as Biomarkers in TC

As we showed above, the special characteristics of circRNA make it an ideal biomarker (Zhang X. et al., 2020). Accumulated evidences show that circRNA is an effective diagnostic and prognostic biomarker of TC. A study showed that hsa_circ_0102272 was associated with poorer clinical characteristics and worse survival data in TC patients (Liu et al., 2020). Hsa_circ_0137287 was reported to be associated with more aggressive clinical characteristics of PTC. Meantime, the study also revealed the value of hsa_circ_0137287 on diagnosing malignancy, extrathyroidal extension, and lymph node metastasis (Lan et al., 2018a). Furthermore, hsa_circ_0011290 was shown to contribute to the progression of PTC and poor prognosis of patients undergoing radical thyroidectomy (Hu et al., 2020). Guo et al. (2020) assessed the associations between circRNA expressions and clinical characteristics in PTC, and some dysregulated circRNAs were found to be associated with BRAFV600E mutation, capsular invasion, advanced T stage, and lymph node metastasis (Guo et al., 2020). The study by Ren et al. (2018) showed that eight dysregulated circRNAs were associated with lymph node metastasis and an advanced TNM stage (Ren et al., 2018). The clinical–pathologically and prognostically related circRNAs are listed in Table 3.

The expression patterns of circRNAs are connected to progression and prognosis of TC, thus having a great value as biomarkers in managing TC patients. Identifying more novel circRNAs with associated clinical features and investigating their biological mechanisms will help us in better recognizing the progression of TC.

## Roles and Significance of circRNAs in TC

### The Underlying Mechanisms of circRNAs

To recognize the specific functions of circRNAs in TC, there are a large number of researches focusing on this issue. Presently, the activities of circRNAs are mainly recognized as the sponging of specific miRNAs, serving as tumor suppressors or promoters. Most of the mechanistic studies on TC-associated circRNAs are designed using a circRNA–miRNA–mRNA axis (Ruan et al., 2020). For instance, circBACH2 acts as a novel oncogenic RNA of PTC. circBACH2, as a miRNA sponge, can negatively mediate miR-139-5p and positively control downstream gene LMO4 expression in PTC cells (Cai et al., 2019). Moreover, hsa_circ_0058124 modulates miRNA-218-5p and its target gene NUMB expression, and consequently with repression of the NOTCH3/GATAD2A signaling axis (Yao et al., 2019). Hou et al. (2018) revealed an association between alkylglycerone phosphate synthase (AGPS) and the malignancy of TC cell lines, and the inactivation of AGPS could decrease the malignancy. Multiple circRNAs were found to be dysregulated after AGPS knock out in PTC cell line, meaning circRNAs could also be correspondingly regulated when gene expression level changed (Hou et al., 2018). Bi et al. (2018) revealed that, by interacting CTNNBIP1, circRNA_102171 could interfere the connection between CTNNBIP1 and the β-catenin/TCF3/TCF4/LEF1 complex, thus activating the Wnt/β-catenin pathway. The direct interaction between circRNA_102171 and CTNNBIP1 has been verified in this study (Bi et al., 2018). circABCB10 expression was significantly higher in TC tissues and the expression of KLF6 was markedly upregulated by the silence of circABCB10, while KLF6 expression was downregulated by overexpression of circABCB10. However, the interaction between circABCB10 and KLF6 has not been experimentally verified (Han et al., 2020). Ma et al. (2020) revealed that circTP53 was highly expressed in TC tissues, and its host gene p53 expression showed a negative association with circTP53 expression via regulating MDM2 mRNA level. Subsequently, knockdown of circTP53 enhanced p53 expression (Ma et al., 2020). Ye et al. (2020) demonstrated that circFOXM1 played a role in PTC by sponging miR-1179 and regulating HMGB1 expression, though the FOXM1 protein was not influenced by the expression of circFOXM1 (Ye et al., 2020). Based on these results, certain circRNA can also regulate its host gene expression in TC.

### Regulation of circRNAs on Proliferation, Migration/Invasion, and Apoptosis of TC

So far, several studies demonstrated that circRNAs served as miRNA sponges and protein interacting molecule in cancer progression by multiple signaling pathways. These circRNAs are activated in TC proliferation, migration/invasion, apoptosis, and chemoresistance. Most studies about circRNAs function focus on these cellular biologic functions. The detailed information about dysregulated circRNAs, targeted miRNA/protein, related signaling pathways, and biologic function is listed in Table 2. circBACH2 (hsa_circ_0001627) was highly expressed in PTC and inhibiting circBACH2 expression in TC cells decreased cell proliferation, migration, and invasion. Mechanistically, the circBACH2 could sponge miR-139-5p, therefore abolishing the downregulated effect on the target gene LMO4, which contributed to TC progression (Cai et al., 2019). circNEK6 and FZD8 were significantly upregulated in TC, with strong correlations. Overexpression of circNEK6 and FZD8 could promote the growth and invasion of TC cells through miR-370-3p and Wnt signaling pathway. Importantly, the effect of miR-370-3p on TC cells could be rescued by circNEK6 or FZD8 (Chen F. et al., 2018). Furthermore, circDOCK1 could increase cyclin D1 and decrease p53 and meanwhile induce the accumulation of MMP-9 and vimentin, thus promoting migration and invasion. circDOCK1 was also reported to contribute to carcinogenesis of TC by inhibiting miR-124 with dampening signaling transduction of JAK/STAT/AMPK (Cui and Xue, 2020). circLDLR was upregulated in PTC, and silence of circLDLR suppressed PTC cell proliferation, migration, and invasion and promoted apoptosis by regulating miR-195-5p/LIPH axis (Gui et al., 2020). The expression of circRNA_102171 was upregulated in PTC tissues and cell lines and the silence of circRNA_102171 suppressed PTC cell proliferation, migration, and invasion and promoted apoptosis by interacting with CTNNBIP1 and activating the Wnt/β-catenin pathway (Bi et al., 2018). On the other hand, hsa_circ_0058124 acts as an oncogene promoting proliferation, tumorigenesis, invasion, and metastasis, by miRNA-218-5p, its target gene NUMB, and the NOTCH3/GATAD2A signaling axis (Yao et al., 2019). A study indicated that the knockdown of circ_0005273 inhibited PTC growth and progression, and further analyses showed that the oncogenic role of circ_0005273 was based on miR-1183 and SOX2 (Zhang W. et al., 2020). In our previous study, we found that hsa_circ_0011385 was associated with TC cell proliferation, migration, invasion, and apoptosis by targeting miR-361-3p (Xia et al., 2020). Other circRNAs involved in TC proliferation, migration/invasion, apoptosis, and chemoresistance can be found in Table 2.

**TABLE 2:** Dysregulated circRNAs in thyroid cancer.

circRNA	Expression change	Function	Targeted gene	Signaling pathway	References.
circBACH2	Upregulated	Proliferation (+) Invasion/migration (+)	miR-139-5p/LMO4	–	Cai et al., 2019
circNEK6	Upregulated	Proliferation (+) Invasion/migration (+) Apoptosis (−)	miR-370-3p/FZD8	Wnt signaling	Chen F. et al., 2018
circDOCK1	Upregulated	Proliferation (+) Invasion/migration (+)	miR-124	–	Cui and Xue, 2020
circLDLR	Upregulated	Proliferation (+) Invasion/migration (+) Apoptosis (−)	miR-195-5p/LDLR	–	Gui et al., 2020
circ_0011290	Upregulated	Proliferation (+) Apoptosis (−) Glycolysis (+)*	miR-1252/FSTL1	–	Hu et al., 2020
circ_0004458	Upregulated	Proliferation (+) Apoptosis (−)	miR-885-5p/RAC1	–	Jin et al., 2018
circITGA7	Upregulated	Proliferation (+) Invasion/migration (+)	miR-198/EGFR1	–	Li S. et al., 2020
circNUP214	Upregulated	Proliferation (+) Invasion/migration (+)	miR-145/ZEB2	–	Li et al., 2018
circRAPGEF5	Upregulated	Proliferation (+) Invasion/migration (+)	miR-198/FGFR1	–	Liu et al., 2019
circTP53	Upregulated	Proliferation (+)	miR-1233-3p/MDM2	P53 signaling	Ma et al., 2020
circ_0025033	Upregulated	Proliferation (+) Invasion/migration (+) Apoptosis (−)	miR-1231 and miR-1340	–	Pan et al., 2019
circ_0058124	Upregulated	Proliferation (+) Invasion/migration (+) Metabolic ability (+) **	miR-940/MAPK1 miR-281-5p/NUMB miR-370-3p/LMO4	NOTCH3 signaling	Yao et al., 2019; Liu et al., 2020; Sun J. W. et al., 2020
circ-ITCH	Downregulated	Proliferation (−) Invasion/migration (−)	miR-22-3p/CBL	Wnt/β-catenin	Wang et al., 2020
circEIF3I (circ_0011385)	Upregulated	Proliferation (+) Invasion/migration (+) Apoptosis (−)	miR-361-3p miR-149/KIF2A	–	Wei et al., 2018; Wang et al., 2020; Xia et al., 2020
circZFR	Upregulated	Proliferation (+) Invasion/migration (+)	miR-1261/C8orf4	–	Wei et al., 2018
circRASSF2	Upregulated	Proliferation (+) Invasion/migration (+) Apoptosis (−)	miR-1178/TLR4	–	Wu et al., 2020a
circFNDC3B	Upregulated	Proliferation (+) Invasion/migration (+)	miR-1178/TLR4	–	Wu et al., 2020b
circPRMT5	Upregulated	Proliferation (+) Invasion/migration (+) Apoptosis (−)	miR-30c/E2F3	–	Xue et al., 2020
circ_0039411	Upregulated	Proliferation (+) Invasion/migration (+) Apoptosis (−)	miR-1179/ABCA9 miR-1205/MTA1	–	Yang Y. et al., 2020
circFOXM1	Upregulated	Proliferation (+)	miR-1179/HMGB1	–	Ye et al., 2020
circ_0067934	Upregulated	Proliferation (+) Invasion/migration (+) Apoptosis (−)	miR-1304/CXCR1	–	Zhang et al., 2019
circ_0005273	Upregulated	Proliferation (+) Invasion/migration (+)	miR-1183/SOX2	–	Zhang Y. et al., 2020
circ_0103552	Upregulated	Invasion/migration (+)	miR-127	–	Zheng et al., 2020
circ_0102272	Upregulated	Proliferation (+) Invasion/migration (+)	–	–	Liu et al., 2020
circ_102171	Upregulated	Proliferation (+) Invasion/migration (+) Apoptosis (−)	CTNNBIP1***	Wnt/β-catenin	Bi et al., 2018
circABCB10	Upregulated	Proliferation (+) Invasion/migration (+)	KLF6****	–	Han et al., 2020
circEIF6	Upregulated	Proliferation (+) Apoptosis (−) Autophagy (+) Chemoresistance (+)	miR-144-3p/TGF-α	–	Liu et al., 2018
circ_0007694	Downregulated	Proliferation (−) Invasion/migration (−) Apoptosis (+)	–	PI3K/AKT/mTOR Wnt signaling****	Long et al., 2020
circ_0067934	Upregulated	Proliferation (+) Invasion/migration (+) Apoptosis (−)	–	PI3K/AKT	Wang et al., 2019
circ_0124055 and circ_0101622	Upregulated	Proliferation (+) Apoptosis (−)	–	–	Sun J. W. et al., 2020
circNCOR2	Upregulated	Proliferation (+) Invasion/migration (+)	miR-516a-5p/MTA2	–	Luan et al., 2020
circHIPK3	Upregulated	Proliferation (+) Invasion/migration (+)	miR-338-3p/RAB23	–	Shu et al., 2020
circ_103598	Upregulated	Proliferation (+)	miR-23a-3p/IL6	–	Zhang Y. et al., 2020
circFAT1 (e2)	Upregulated	Proliferation (+) Invasion/migration (+)	miR-873/ZEB1	–	Liu et al., 2020a

*Suppressed glucose uptake, decreased lactate production, and increased ATP contents were associated with Has_circ_0011290-depletion. **The oxygen consumption rate (OCR) of basal respiration and maximum respiration in TPC cells was significantly decreased after circ_0058124 knockdown. ***The direct interaction was verified by RNA pulldown. ****The interaction between Circ-ABCB10 and KLF6 was only bioinformatically predicted.

**TABLE 3:** CiRNAs and TC diagnosis and prognosis.

circRNA	No. of samples	Tumor size	Lymph node metastasis	Extrathyroidal extension	Mutifocality	M stage	TNM stage I/II VS. III/IV	Survival rate	References
circBACH2	40	≤ 1 cm vs. ≥ 1 cm (+)	+	+	NA	NA	+	OS (+)	Cai et al., 2019
circ_0011290	40	NA	NA	NA	NA	NA	+	OS (+)	Hu et al., 2020
circ_0004458	48	≤ 3 cm vs. ≥ 3 cm (+)	+	+	NA	+	+	NA	Jin et al., 2018
circ_0058124	51	NA	NA	NA	NA	NA	+	NA	Sun J. W. et al., 2020
circ_0058124	92	≤ 1 cm vs. ≥ 1 cm (+)	+	+	−	+	+	NA	Yao et al., 2019
circITCH	37	NA	+	NA	NA	NA	+	Prognostic (+) *	Wang et al., 2018
circZFR	41	NA	+	NA	NA	NA	+	OS (+)	Wei et al., 2018
circRASSF2	112	−	+	NA	−	NA	+	NA	Wu et al., 2020a
circFNDC3B	42	≤ 1 cm vs. ≥ 1 cm (+)	+	NA	−	NA	+	OS (+)	Wu et al., 2020b
circPRMT5	55	NA	+	NA	NA	NA	+	NA	Xue et al., 2020
circFOXM1	78	≤ 3 cm vs. ≥ 3 cm (+)	+	NA	NA	NA	+	NA	Ye et al., 2020
circ_00067934	50	NA	+	NA	NA	NA	+	OS (+)	Zhang et al., 2019
circ_0005273	50	NA	NA	NA	NA	NA	NA	OS (+)	Zhang Y. et al., 2020
circ_0137287	120	≤ 2 cm vs. ≥ 2 cm (+)	+	+	−	NA	−	NA	Lan et al., 2018a
circ_0102272	58	≤ 2 cm vs. ≥ 2 cm (−)	+	NA	NA	NA	+	OS (+) DFS (+)	Liu et al., 2020a
circ_0067934	57	≤ 1 cm vs. ≥ 1 cm (+)	+	NA	NA	NA	+	Prognostic (+) *	Wang et al., 2019
circ_047771	40	−	+	NA	−	NA	+	NA	Ren et al., 2018
circ_007148	40	−	−	NA	−	NA	+	NA	Ren et al., 2018
circ_0124055	66	≤ 2 cm vs. ≥ 2 cm (+)	+	NA	NA	NA	+	OS (+)	Sun J. W. et al., 2020
circ_0101622	66	≤ 2 cm vs. ≥ 2 cm (+)	+	NA	NA	NA	+	OS (+)	Sun J. W. et al., 2020
circ_103598	100	≤ 5 cm vs. ≥ 5 cm (+)	NA	NA	NA	+	+	OS (+)	

*It has not been specified in these research that circ-ITCH is an independent prognostic marker with OS rate or DFS rate.

### Regulation of circRNAs on Glucose Metabolism and Metabolic Ability

Hu et al. (2020) revealed hsa_circ_0011290 acting as an oncogene in PTC; meantime, glucose metabolism was changed to decreased glucose uptake and lactate production and increased ATP contents, which was proven to be associated with hsa_circ_0011290 via miR-1252. They also confirmed that the downstream targets of hsa_circ_0011290 were miR-1252 and FSTL1. These data highlighted the importance of the metabolic profiling of hsa_circ_0011290 in PTC (Hu et al., 2020). Sun D. et al. (2020) revealed that circ_0058124 promoted proliferation, migration, and invasion in TC cells with miR-940/MAPK1 as the downstream targets. In addition, after knocking circ_0058124 down, the oxygen consumption rate (OCR) of basal respiration and maximum respiration significantly decreased via miR-940, indicating inhibited metabolic ability of cells. Subsequently, miR-940 inhibitor rescued the inhibition effect of silencing MAPK1 on the metabolic ability. Therefore, these results confirmed the regulatory role of circ_0058124 on metabolisms via the miR-940/MAPK1 axis in TC (Sun D. et al., 2020).

### Regulation of circRNAs on Chemoresistance

Liu et al. (2018) revealed that, after the therapy of cisplatin (30 μg/ml), the expression of circEIF6 was continuously increased and reached a highest level in 24 h. Given the oncogenic role of circEIF6 via miR-144-3p/TGF-α, circEIF6 could develop cisplatin resistance by miR-144-3p/TGF-α in TC cells. They also demonstrated that circEIF6 knockdown increased cisplatin sensitivity in vivo. However, no evidence showed a difference between control group and circEIF6 knockdown group (silencing by targeted shRNA). Similarly, circEIF6 knockdown effectively reduced tumor weight in the nude mice treating with cisplatin, while circEIF6 knockdown alone had no effect on tumor weight (Liu et al., 2018). Moreover, Zhang S. et al. (2020) revealed that circRNA_103598 increased as a regulator increasing the expression of IL-6 via sponging miR-23a-3p in PTC, with promoted cell proliferation and the enhanced oncolytic vaccinia virus (OVV)-mediated antitumor effect by strengthening the viral replication.

Although many circRNAs are revealed as functioning in TC development, most of these studies are performed mainly within cell line level. Further studies with well-established animal models, including gene knockout models, are needed for the preparation of conversions to clinical application. In addition, the effects of circRNAs on chemoresistance are very inspiring, for the difficult problem of treating TC patients with chemoresistance. Besides understanding the role of circRNAs on chemoresistance, novel circRNA-related therapy targets may also offer help to this difficult problem.

### circRNAs in Serumal Exosomes and Serum

Exosomes are small endocrine vesicles that exist in most cells and contain transporters, mRNA, and miRNA. In exosomes, these molecules have a potential role as diagnostic biomarkers of human disease. Recent studies showed that there were many circRNAs existing in exosomes, and a study further showed the expression of circRNAs in exosomes was associated with that in cells and tissues (Yang et al., 2019). Yang et al. (2019) have isolated the exosomes and associated circRNAs were analyzed by high-throughput sequencing. Three upregulated and 19 downregulated circRNAs were found, and three dysregulated circRNAs, including hsa_circ_007293, hsa_circ_031752, and hsa_circ_020135, were finally confirmed by experiments (Yang et al., 2019). Zhou et al. (2019) have reported that circRASSF2 mediated tumor progression through the miR-1178/TLR4 pathway in PTC. They also found a negative association between circRASSF2 and miR-1178 in serum exosomes among PTC patients (Wu et al., 2020a). After verifying the role of circFNDC3B in modulating PTC progression, Wu et al. (2020b) have also collected exosomes from 42 PTC patients and 40 healthy people. They revealed that upregulated circFNDC3B expression could be detected in extracted serum exosomes derived from PTC patients (Wu et al., 2020b).

Fan et al. (2019) demonstrated that circMAN1A2 was highly expressed in sera of TC patients, and diagnostic ROC curves showed that circMAN1A2 had the potential being a diagnostic biomarker. Sun J. W. et al. (2020) measured the expression levels of hsa_circ_0124055 and hsa_ circ_0101622 in plasma from 65 TC patients and 65 healthy volunteers. The expression levels of hsa_circ_0124055 and hsa_circ_0101622 were observed to be significantly upregulated in the plasma of TC patients. To explore whether plasma circRNAs were susceptible to neoplasm growth, qRT-PCR analysis was carried out to detect hsa_circ_0124055 and hsa_circ_0101622 levels in the plasma of TC patients before and 14 days after surgery. Noteworthily, their findings uncovered the significant decline of hsa_circ_0124055 and hsa_circ_0101622 in plasma of TC patients with thyroidectomy (Sun J. W. et al., 2020). Shi et al. (2020) revealed circRAPGEF5 and hsa_circ_0058124 were dysregulated in serum of PTC patients with further analyses showing that these two circRNAs significantly decreased after systematic treatments. Yu et al. (2020) revealed that combination of circRNA-UMAD1 and Gal3 from peripheral circulation was a useful and effective co-biomarker for the prognosis of LNM in PTC patients.

The findings from these studies have shown that exosome and serumal circRNAs can be obtained and detected from TC patients, supporting future studies on the role of exosome and serumal circRNAs in TC. In fact, exosome, as a natural mediator, might greatly extend circRNAs studies and applications in TC. It has also been considered as a suitable drug delivery tool. Modification of exosome sheds light on a novel direction for TC diagnosis and treatment and, therefore, brings the great potential of exosomal circRNA as a remarkable biomarker and therapy tool for TC.

## Conclusion and Future Perspectives

With the growth of findings and progresses in the field of RNA, circRNA has gradually become a hot spot in tumor researches. Microarray profiling and high-throughput RNA sequencing facilitate the discovery of circRNA transcripts in cancers, including TC. Researches have revealed that circRNA can serve as a regulatory molecule involved in various biological processes. This review highlights the expression of circRNAs in TC, burgeoning the potential of circRNA as a regulatory molecule, diagnostic, prognostic, and therapeutic tool in TC research. circRNAs are involved in multiple intricate functions and molecular mechanisms; however, only a small fraction of circRNAs and their functions in TC biology are found and researched. In addition, the functions of the few known circRNAs still need to be investigated in detail and additional functional circRNAs remain to be identified. A great number of problems remain when it comes to the biogenesis, diagnostic application, and function of circRNAs in TC. For example, there is still no relevant study on the abnormal regulation of circRNA expression level in fine-needle aspiration (FNA) biopsies of TC, while 20% of FNA biopsies show undetermined pathological results, leading to the poor management for these patients. Although most PTC can be effectively controlled, mortality associated with advanced and iodine-refractory TC is remaining as a high level. Meanwhile, ATC represents extremely poor prognosis. Whether circRNAs can be used in understanding the pathogenesis or serve as biomarkers for these advanced TC early diagnosis and effective treatment need to be further studied. Epigenetic circRNA regulation has an extremely great potential in managing cancers including TC and should be focused by future studies for not only acquiring a better understanding of TC but also achieving therapeutic successes eventually.

## Author Contributions

XL, FX, and ZZ conceived of the study. FX and ZZ drafted the manuscript. ZZ and XL edited the manuscript. All authors read and approved the final manuscript, and agreed to be accountable for all aspects of the work in ensuring that questions related to the accuracy or integrity of any part of the work are appropriately investigated and resolved.

## Conflict of Interest

The authors declare that the research was conducted in the absence of any commercial or financial relationships that could be construed as a potential conflict of interest.
